# Genetic Variants in the Transcriptional Regulatory Region of the ALOX5AP gene and Susceptibility to Ischemic Stroke in Chinese Populations

**DOI:** 10.1038/srep29513

**Published:** 2016-07-15

**Authors:** Dongzhi Yang, Xiangnan Huang, Chuanju Cui, Yuchao Zhang, Ya Li, Xin Zang, Ying He, Hong Zheng

**Affiliations:** 1 School of life sciences of Zhengzhou University, Zhengzhou, 450052, China; 2 Department of Neurology, The First People’s Hospital of Zhengzhou, Zhengzhou, 450000, China; 3 Department of Medical Genetics & Cell Biology, School of Basic Medical Sciences, Zhengzhou University, Zhengzhou, 450052, China

## Abstract

No coding sequence variants of the *ALOX5AP* gene that lead to amino acid substitutions have been identified. A two-stage study design was used to explore the relationship between variants in the transcriptional regulatory region of *ALOX5AP* gene and ischemic stroke (IS) risk in Chinese populations. IS was determined using CT and/or MRI. First, 18 SNPs, located in the upstream promoter region of *ALOX5AP* gene, were genotyped in 200 IS patients and 200 controls. And one potential associated SNP (rs17222919) was identified (*P* = 0.005,*OR = 0.623*, 95% *CI*: 0.448~0.866). Next, another independent case-control cohort comprising 810 IS patients and 825 matched controls was recruited to investigate the role of rs17222919, rs9579646 polymorphisms and their haplotypes in IS risk. The G allele frequency of rs17222919 in the IS group was significantly lower than that in control group (*P* = 0.007, *OR* = 0.792, 95% *CI*: 0.669~0.937). T-A and G-A haplotypes were associated with IS (*P* = 0.001,*OR* = 1.282, 95% *CI*:1.100~1.495; *P* = 0.0001, *OR* = 0.712, 95% *CI*: 0.598~0.848; respectively). Our study providesevidence that rs17222919 is a potential genetic protective factor against IS. Furthermore, the T-A haplotype is a risk factor and the G-A haplotype is a protective factor against IS in Chinese population.

Ischemic stroke (IS), also called cerebral infarction (CI), is a complex multifactorial disorder characterized by the sudden loss of blood circulation to an area of the brain, resulting in a corresponding loss of neurologic function^1^. Previous studies have suggested that inflammation is a key element in all critical steps of atherosclerosis, which underlies the pathogenesis of cardiovascular disease and IS^2^. The 5-lipoxygenase-activating protein (FLAP), which is encoded by the *ALOX5AP* gene, is a crucial regulator of the biosynthesis of leukotrienes (LTs), which can lead to the accumulation of LTs in fatty deposits on the arterial wall^3^. LTs initiate leukocyte activation and promote the adhesion of monocytes on the vascular wall, a process that plays an important role in the pathogenesis of atherosclerosis and inflammatory diseases, including IS^4^.

To date, most genetic studies have focused on the relationship between two at-risk haplotypes (HapA and HapB) of the *ALOX5AP* gene and the susceptibility to IS. However, the association results were inconsistent and controversial across different ethnic backgrounds^5^,^6^,^7^. In particular, no coding sequence variants of the *ALOX5AP* gene that lead to amino acid substitutions have been identified^8^. Domingues-Montanari *et al*.^9^ reported that the SG13S114 genotypes modulate the mRNA levels of *ALOX5AP* gene and the mRNA levels were higher in IS cases than in controls. Helgadottir *et al*.^10^ revealed that the *ALOX5AP* gene expression levels and its downstream leukotriene B_4_ (LTB_4_) synthesis activity were greater in IS patients than in controls. Kim *et al*.^11^ previously reported that a promoter polymorphism (rs17222919) was associated with the development of intracerebral hemorrhage in the Korean population. Ji RJ *et al*.^12^, also reported that another promoter polymorphism (−581_582 Ins A) might be a novel genetic risk factor for IS in a north Chinese Han population. These phenomena strongly suggested that there might be some additional unidentified variants in the regulatory regions of the *ALOX5AP* gene may play modulate IS risk. However, the relationship between polymorphisms throughout the entire transcriptional regulatory region of the *ALOX5AP* gene and IS risk has not been extensively explored.

Therefore, a two-stage study design was used to explore the relationship between variants of *ALOX5AP* gene and IS risk in two independent Chinese Han cohorts. Firstly, we selected 18 SNPs that cover the promoter region of the *AlOX5AP* gene to screen the positive SNPs using SNaPshot minisequence technique. Subsequently, we investigated the role of rs17222919 (in the promoter region) and rs9579646 (in the first intron region) polymorphisms in IS risk using TaqMan-PCR technique in a larger cohort from the Chinese Han population.

## Results

### Subject characteristics

The clinical and demographic characteristics of the two populations are shown in Table 1. Cases and controls were well matched in age and sex (*P* > 0.004; *P* > 0.025). Compared with the control groups, the IS groups showed higher percentages of hypertension, diabetes mellitus and smoking (*P* < 0.004; *P* < 0.025). IS patients also had significantly higher total cholesterol (TC) and total triglycerides (TG) levels than the control subjects ((*P* < 0.004; *P* < 0.025).

### The preliminary screening results in the initial study

#### Association analysis of 18 SNPs in the promoter region of ALOX5AP with IS

The −1785G>A, −946A>G, −581_582lnsA, −519G>A, −290G>A and −190G>A were not polymorphic in our initial study. The genotype distributions of rs12560847, rs9578195, rs61947373, rs34404999, rs55950839, rs55780307, rs59227506, rs34536374, rs34344566, rs9578194 and rs34352240 were consistent with the Hardy–Weinberg equilibrium, but showed no significant differences between IS patients and controls (*P* >** **0.004).

For the rs17222919 polymorphism, the G allele frequency was significantly lower in IS group (19.5%) than in control group (28.0%) (*P* = 0.005; *OR = *0.623, 95% *CI*: 0.448~0.866). See Table 2.

#### Haplotype analysis of the ALOX5AP promoter region

**Figure 1:**
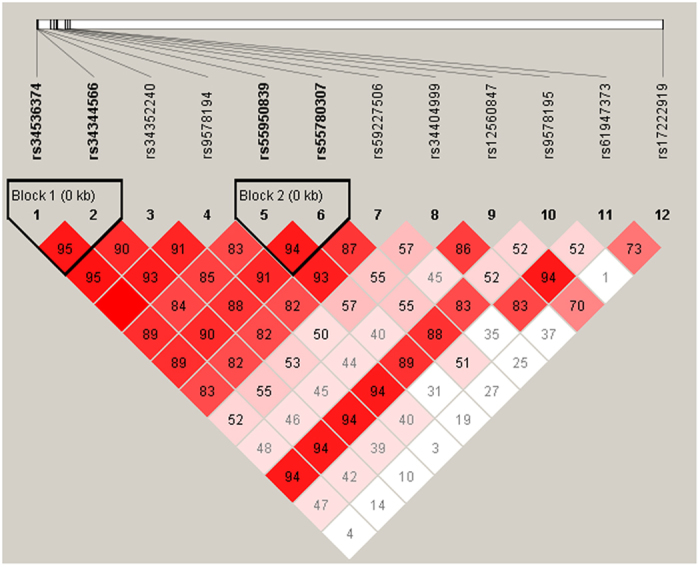
The results of linkage disequilibrium tests for the twelve analyzed SNPs (The order is rs34536374, rs34344566, rs34352240, rs9578194, rs55950839, rs55780307, rs59227506, rs34404999, rs12560847, rs9578195, rs61947373 and rs17222919 from left to right).

After the non-polymorphic loci were removed, linkage disequilibrium (LD) test was performed and the results were shown in Fig. 1. The rs34536374, rs34344566, rs34352240, rs9578194, rs55950839, rs55780307, rs59227506 and rs12560847 were in strong linkage disequilibrium and haplotype blocks (*D’* > 0.8) were defined. Because rs17222919 was not in strong linkage disequilibrium (*D’*<0.8), the haplotype blocks were defined without rs17222919. The distribution of haplotype (CACGTATG) and haplotype (TGTAAGCA) has no significant difference between IS group and control group (*P* = 0.037). See Table 3.

### Association between the rs17222919 and rs9579646 polymorphisms and IS in the second cohort

#### Association analysis and inherited model test

The genotype frequency distributions of rs17222919 and rs9579646 were consistent with Hardy-Weinberg equilibrium (HWE) in the second cohort. After adjusting for conventional risk factors, the G allele frequency (19.3%) in the IS group was found to be significantly lower than that (23.2%) in the control group (*P* = 0.007, *OR* = 0.792, 95% *CI* = 0.669~0.937). See Table 4. However, the rs9579646 polymorphism showed no significant differences between the two groups (*P* > 0.025).To assess the effect of rs17222919 on the risk of IS, we compared additive, dominant, and recessive models. The effect of rs17222919 was best described with the dominant model (*P = *0.016 *OR* = 0.778, 95% *CI* = 0.637~0.951). See Table 5.

#### Linkage disequilibrium test and haplotype analysis

Linkage disequilibrium test indicated that rs17222919 and rs9579646 were in strong linkage disequilibrium (*D’* = 0.837). Consequently, haplotype analysis was carried out. Three common haplotypes (frequency >3%) in the cases and controls were presented in Table 6. There was a statistically significant difference in the frequency of the G-A haplotype between the case and control groups (*P* = 0.0001, *OR* = 0.712, 95% *CI*: 0.598–0.848). The frequency of the T-A haplotype was higher in the IS group than in the control group (*P* = 0.001, *OR = *1.282, 95% *CI*: 1.100~1.495).

## Discussion

Promoter elements have fundamental roles in regulating gene transcription. Altering the DNA sequence of a promoter may result in changes in transcription factor binding sites or binding rates. Thus, a single nucleotide polymorphism (SNP) in the promoter region can affect promoter activity by altering the binding affinity of transcription factors involved in the regulation of gene expression^13^,^14^. In the present study, we designed a two-stage study to explore the relationship between variants in the transcriptional regulatory region of the *ALOX5AP* gene and IS risk. To the best of our knowledge, this is a comprehensive study to evaluate whether polymorphisms in the transcriptional regulatory region of *ALOX5AP* gene could influence susceptibility to IS in a large Han Chinese population.

Firstly, 18 SNPs that cover the promoter region of the *ALOX5AP* gene were selected using SNaPshot method to screen positive SNPs. The results showed that −1785G>A, −946A>G, −581_582lnsA, −519G>A, −290G>A and −190G>A loci were not polymorphic in our initial population. Our results were obviously different from those reported by Ji RJ *et al*.^12^, who reported that three genetic variants were identified, including a mutation (−519 G/A), an insertion and deletion polymorphism (−581_582 Ins A) and a single nucleotide polymorphism (−946 A/G). They also reported that the −581_582 Ins A polymorphism might be a novel genetic risk factor for IS in a north Chinese Han population. The discrepancy between their findings and those of our study may be attributed to the differences in sample size. Therefore, further study with a larger population is required to confirm the findings. Next, the genotype distributions of 11 SNPs, including rs12560847, rs9578195, rs61947373, rs34404999, rs55950839, rs55780307, rs59227506, rs34536374, rs34344566, rs9578194 and rs34352240 were consistent with the Hardy–Weinberg equilibrium but showed no significant differences between IS and controls (*P* > 0.004). However, the G allele frequency of rs17222919 was significantly different between the IS and control groups (*P* = 0.005; *OR = 0.623*, 95% *CI*: 0.448~0.866). If the adjusted *P* value for significance was set at 0.05/12 = 0.004 in the initial cohort by Bonferroni’s adjustment, the *P* value of rs17222919 (0.005) was close to the statistical significance. So we thought it was a potentially associate locus.Therefore, the rs17222919 was identified to have a potentially positive association with IS in the first-stage study and was to be replicated in a larger Chinese Han cohort.

Haplotype analysis is considered more powerful than single SNP analysis to search for genetic determinants of complex diseases^15^. After the non-polymorphic loci were excluded, LD test was performed. The results indicated that rs34536374, rs34344566, rs34352240, rs9578194, rs55950839, rs55780307, rs59227506 and rs12560847 were in strong linkage disequilibrium (*D’* > 0.8) and haplotype blocks were defined. The 8 SNPs constituted a total of 22 common potential haplotypes in the patients and controls. And the most frequent haplotype was Hap(CACGTATG). The frequency of haplotype (CACGTATG) was significantly higher in the IS patients than in controls (*P* = 0.037,*OR = 1.540*, 95% *CI*:1.024~2.314). The frequency of haplotype (TGTAAGCA) was significantly lower in the IS patients than in controls (*P* = 0.037,*OR = 0.650*, 95% *CI*:0.432~0.976). However, both of the two haplotypes didn’t reach the statistical significance. Further study with a larger population is required to evaluate the findings.

Preliminary studies have revealed that the introns have important biological functions. In particular, the first introns of human genes are likely to be involved in transcriptional regulation^16^,^17^. Thus, in the second larger Chinese cohort, we investigated the role of rs17222919 (in the promoter region) and rs9579646 (in the first intron region) polymorphisms in IS risk using TaqMan-PCR technique. For the rs17222919 polymorphism, the G allele frequencies of IS group (19.3%) were significantly lower than in the control group (23.2%) (*P = *0.007). Multivariate logistic regression analysis showed that the G allele was associated with a 0.792-fold increased risk of IS after adjusting for conventional risk factors (95% *CI*, 0.669~0.937; *P = *0.007). Rs17222919 was associated with IS in a dominant genetic model (*P < *0.025) after performing genotype association tests with dominant, recessive and additive models. However, this association was inconsistent with the results previously reported by Kim *et al*.^11^, who showed that rs17222919 was associated with intracerebral hemorrhage, but not IS, in a Korean population. Our previous i*n vitro* promoter assay revealed that the G allele had a lower transcriptional activity than the T allele, suggesting that the −1316T/G variation reduces *ALOX5AP* promoter activity and consequently down-regulates gene expression. The decreased *ALOX5AP* transcription results in increased inactivation of the 5-LO pathway and reduces leukotriene biosynthesis, which in turn protects from IS^18^. The rs9579646 polymorphism showed no significant differences between groups and was not associated with IS in our second Chinese cohort.

Finally, the linkage disequilibrium test and haplotype analysis were performed using SHEsis software. The rs17222919 and rs9579646 polymorphisms were in complete linkage disequilibrium (*D’* = 0.837). The frequency of the G-A haplotype was lower in the IS group than in the controls (*P* = 0.0001), suggesting that the G-A haplotype might be a genetic protective factor against IS in the Chinese Han population (*OR = *0.712, 95% *CI*: 0.598–0.848). Conversely, the T-A haplotype was associated with an increased risk of IS (*OR* = 1.282, 95% *CI:*1.100~1.495) and the T-A haplotype might be a risk factor for IS in this Chinese Han cohort. Furthermore, the G-G haplotype frequencies were quite low, and this haplotype thus had less impact on the incidence of IS.

Several limitations of our study need to be addressed. First, the sample size of the initial study (200 cases and 200 controls) may not be sufficiently large to screen all the potential risk-associated SNPs and evaluate gene–environment interactions. Second, there was potential selection bias because the cases and controls were recruited from hospital. Third, *ALOX5AP* mRNA levels among different rs17222919 genotypes were not compared in either the IS or control group.

In conclusion, we designed a two-stage study to explore the relationship between variants in transcriptional regulatory region of the *ALOX5AP* gene and IS. In the first stage, 18 SNPs covering the promoter region of the *ALOX5AP* gene were screened and one potential risk-associated SNP (rs17222919) was selected. In the second larger Chinese cohort, we confirmed that the rs17222919 polymorphism was associated with a decreased risk of IS. In addition, two haplotypes were discovered to be associated with IS. Future studies of the variants in the transcriptional regulatory region of *ALOX5AP* and their biological functions should be conducted to further elucidate the etiology of IS.

## Methods

### Study populations

In the initial study, 200 IS patients (112 men and 88 women, mean age 57.2 ± 7.2 years) were recruited from the First Affiliated Hospital of Zhengzhou University. In the second cohort, a total of 810 patients with ischemic stroke (males: females = 416:394, mean age 57.7 ± 8.6 years) were enrolled from Henan Provincial Hospital in central China. None of the participants were included in both populations. The IS was defined by a loss of global or focal cerebral function persisting for >24 h with corresponding infarction on brain imaging with a probable vascular cause^19^. IS cases were classified into three subtypes, namely large-artery atherosclerosis (LAA), small-artery occlusion lacunar (SAO), and stroke of other undetermined etiology (SUE), according to the Trial of Org 10172 in Acute Stroke Treatment (TOAST)^20^. Brain imaging was carried out using computed tomography (CT) and/or magnetic resonance imaging (MRI) as well as ancillary diagnostic investigations and standardized blood tests were also performed. Patients with atrial fibrillation, cerebral hemorrhage, peripheral vascular diseases, or kidney diseases were excluded from the study.

The control groups consisted of 200 (initial study: 106 men and 94 women, mean age 55.9 ± 7.0 years) and 825 (second study: 428 men and 397 women, mean age 55.3 ± 7.2 years) unrelated Henan Han individuals, selected from the same demographic area and matched to the cases by age, sex, and residency. All controls were free of cerebrovascular disease, cardiovascular disease, hepatic disease, renal disease and cancer.

The study protocols were approved by the Ethics Committee on Human Research of Zhengzhou University and informed written consent was obtained from each participant. All experiments were performed in accordance with relevant guidelines and regulations.

### Preliminary screening for positive SNPs by SNaPshot

Because promoter elements have key roles in regulating gene transcription, we selected 18 SNPs covering the promoter region of *ALOX5AP* gene for initial analysis. The −1785G>A, −946A>G, −581_582lnsA, −519G>A, −290G>A and −190G>A were selected based on previously reported significant associations. The rs12560847, rs9578195, rs61947373, rs17222919, rs34404999, rs55950839, rs55780307, rs59227506, rs34536374, rs34344566, rs9578194 and rs34352240 were selected based on pairwise *r*^*2*^ (>0.8) among all common SNPs with minor allele frequency (MAF) >0.1 spanning the promoter region of the *AlOX5AP* gene using the Haploview 4.0 software^21^.

Genomic DNA was extracted from peripheral white blood cells of 200 pairs case and control participants using the Blood Genomic DNA Miniprep Kit (Axygen Biotechnology, Union City, CA, USA) according to the manufacturer’s instructions. Touch-down PCR amplifications was performed using 7 pairs of primers (Sangon, Shanghai, China). All primers were designed using the primer3 program (http://frodo.wi.mit.edu/cgi-bin/primer3/primer3_www.cgi). All samples were genotyped using SNaPshot minisequence technique.

A 10 μL mixture containing 1x HotStarTaq buffer, 3.0 mM Mg^2+^, 0.3 mM dNTP, 1U HotStarTaq polymerase (Qiagen), 1 μL multiple PCR primers and 1 μL template DNA was prepared for each reaction. The cycling program was 95 °C for 2 mins; 11 cycles of 94 °C for 20 s, (62 °C −0.5 °C/cycle) for 40 s, 72 °C for 1.5 mins; 24 cycles of 94 °C for 20 s, 57 °C for 30 s, 72 °C for 1.5 mins; and 72 °C for 2 mins. To eliminate excess primers and dNTPs, 5U SAP (Promega, USA) and 2U Exo I (Epicentre, Palmerston North, New Zealand) were added to the 10 μL PCR products. The mixture was incubated at 37 °C for 60 mins, followed by incubation at 75 °C for 15 mins.

Eighteen extension primers were designed for multiplex PCR SNaPshot reaction. The reaction mixture included 5 μL SNaPshot Multiplex reaction mix (Applied biosystems, USA), 2 μL ddH_2_O, 1 μL extension primer mix and 2 μL purified PCR product. The cycling program was 96 °C for 1 min; 28 cycles of 96 °C for 10 s, 55 °C for 5 s and 60 °C for 30 s. In order to purify the extension products, 1U SAP (Promega, USA) was added to extension product and incubated at 37 °C for 60 mins, followed by incubation at 75 °C for 15 mins. Finally, a mixture of 9 μL HiDi Formamide, 0.5 μL Liz120 size standard and 1 μL purified extension product were denatured at 95 °C for 5 mins and then loaded onto an ABI3730xl instrument (ABI, USA) and GeneMapper4.1 (Applied biosystems, USA) was run to analyze the results.

### Genotyping of rs17222919 and rs9579646 polymorphisms

The preliminary screening results showed that only rs17222919 in the *ALOX5AP* gene promoter region had a significantly different frequency in the IS and control groups. Moreover, the first introns of human genes are likely to be involved in transcriptional regulation. Thus, in the second cohort, we selected rs17222919 which was positively associated with IS in the preliminary study and rs9579646 which is located in the first intron of the *ALOX5AP* gene for analysis.

EDTA anti-coagulated venous blood samples were collected from the 810 enrolled IS patients and 825 healthy controls. Genomic DNA was extracted from the peripheral blood using the Blood Genomic DNA Miniprep Kit (Axygen Biotechnology, Union City, CA, USA). TaqMan probes were used to analyze rs17222919 and TaqMan-MGB probes were selected to genotype rs9579646 polymorphism respectively. The genotype was determined according to the relative fluorescence intensity of the probe detected by the real-time PCR system (ABI PRISM 7500; Applied Biosystems, Foster City, CA, USA). Each PCR reaction mixture (10 μL) contained 5 μL of 2× TaqMan Universal PCR Master Mix (Applied Biosystems), 0.2 μL forward primer, 0.2 μL reverse primer, 0.6 μL FAM-labeled probe, 0.6 μL HEX-labeled probe, 0.4 μL ROXII, 1.0 μL of DNA template (1~20 ng/μL), and 2 μL ddH_2_O. In each assay, three samples with known genotypes and three no-DNA blank controls were included. The PCR reaction conditions consisted of pre-degeneration for 2 mins at 95 °C, followed denaturation at 95 °C for 15 s, and 40 cycles of annealing and extension at 60 °C for 30 s. After PCR amplification, sample genotypes were determined by measuring the allele-specific fluorescence with an ABI Prism 7700 Sequence Detection System and using SDS 1.7 software for allele discrimination (Applied Biosystems). To verify the genotyping accuracy, 10% of random samples were sequenced.

### Statistical analysis

All statistical analysis was performed using the SPSS 17.0 package (SPSS Inc., Chicago, IL, USA). Pearson’s chi-squared test was used to test for differences in qualitative variables and genotype/allele frequencies. Differences in quantitative variables between groups were analyzed using Student’s *t*-test. Testing for deviation of genotype distribution from Hardy–Weinberg equilibrium and a haplotype-based case-control study were performed using SHEsis software (http://analysis.bio-x.cn)^22^. Odds ratios (*OR*s), 95% confidence intervals (95% *CI*s) and corresponding *P* values for IS risk were calculated by logistic regression analysis after adjusting for age, gender, hypertension, diabetes, smoking, drinking and biochemical indexes such as TC and TG levels. Bonferroni’s adjustment was used for multiple comparisons. The adjusted *P* value for significance was set at 0.05/12 = 0.004 in the initial cohort and the adjusted *P* value less than 0.025 were considered statistically significant in the second cohort.

## Additional Information

**How to cite this article**: Yang, D. *et al*. Genetic Variants in the Transcriptional Regulatory Region of the ALOX5AP gene and Susceptibility to Ischemic Stroke in Chinese Populations. *Sci. Rep.* **6**, 29513; doi: 10.1038/srep29513 (2016).

## Figures and Tables

**Table 1:** Characteristics of the two independent study populations.

	Initial study Population			Second study Population		
	Cases(n = 200)	Controls(n = 200)	*P*value	Cases(n = 810)	Controls(n = 825)	*P*value
Sex (males/females)	112/88	106/94	0.547	416/394	428/397	0.833
Age (mean ± SD, years)	57.2 ± 7.2	55.9 ± 7.0	0.090	57.7 ± 8.6	55.3 ± 7.2	0.243
Total cholesterol (mmol/L)	5.18 ± 1.09	4.54 ± 1.34	0.000*	5.22 ± 1.17	4.62 ± 1.29	0.016*
Total triglyceride (mmol/L)	1.89 ± 1.03	1.41 ± 1.01	0.000*	1.94 ± 1.31	1.35 ± 1.13	0.017*
Hypertension, (n, %)	80 (40.0)	26 (13.0)	0.000*	469 (57.9)	98 (11.9)	0.000*
Diabetes, (n, %)	32 (16.0)	9 (4.5)	0.000*	134 (16.5)	26 (3.2)	0.000*
Smokers, (n, %)	33 (16.5)	20 (10.0)	0.055	112 (13.8)	64 (7.8)	0.000*
Alcohol, (n, %)	34 (17.0)	28 (14.0)	0.407	133 (16.4)	118 (14.3)	0.235

*P < 0.004 denotes statistical significance in the initial cohort.

*P < 0.025 denotes statistical significance in the second cohort.

**Table 2:** The genotype and allelic distribution of 18 SNPs in the promoter region of *ALOX5AP* in IS and control subjects.

SNP	Allele	Cases (n/%)	Controls (n/%)	*P* value	*OR* (95%*CI*)
1785G>A	G	400	400		
	A	0	0	—	—
946A>G	A	400	400		
	G	0	0	—	—
581_582lnsA	D	400	400		
	I	0	0	—	—
519G>A	G	400	400		
	A	0	0	—	—
290G>A	G	400	400		
	A	0	0	—	—
190G>A	G	400	400		
	A	0	0	—	—
rs34536374	C	331 (82.75)	316 (79)		
	T	69 (17.25)	84 (21)	0.178	0.784 (0.551–1.117)
rs34344566	G	74 (18.5)	88 (22)		
	A	326 (81.5)	312 (78)	0.218	1.243 (0.879–1.756)
rs9578194	G	322 (80.5)	306 (76.5)		
	A	78 (19.5)	94 (23.5)	0.169	0.789 (0.562–1.106)
rs34352240	T	70 (17.5)	88 (22)		
	C	330 (82.5)	312 (78)	0.110	1.330 (0.937–1.887)
rs34404999	A	62 (15.5)	80 (20)		
	C	338 (84.5)	320 (80)	0.096	1.363 (0.946–1.964)
rs55950839	T	327 (81.75)	311 (77.75)		
	A	73 (18.25)	89 (22.25)	0.159	0.780 (0.552–1.103)
rs55780307	A	340 (85)	325 (81.25)		
	G	60 (15)	75 (18.75)	0.157	0.765 (0.527–1.109)
rs59227506	T	330 (82.5)	319 (79.75)		
	C	70 (17.5)	81 (20.25)	0.320	0.835 (0.586–1.191)
rs12560847	C	180 (45)	204 (51)		
	T	220 (55)	196 (49)	0.089	1.272 (0.963–1.680)
rs9578195	G	332 (83)	315 (78.75)		
	A	68 (17)	85 (21.25)	0.126	0.759 (0.533–1.082)
rs61947373	G	231 (57.75)	253 (63.25)		
	A	169 (42.25)	147 (36.75)	0.112	1.259 (0.948–1.673)
rs17222919	T	322 (80.5)	288 (72)		
	G	78 (19.5)	112 (28)	0.005*	**0.623 (0.448**–**0.866)**

*P* value and *OR* (95% *CI*) were adjusted for confounding factors such as age, gender, hypertension, diabetes, smoking, drinking, TC and TG. Adjusted *P* value (*P* < 0.004) indicates statistical significance.

**Table 3:** Comparison of haplotype frequencies of eight SNPs excluding rs17222919 between IS and controls.

Haplotype	IS group	Control group	χ^2^	*P*value	*OR*(95% CI)
	(2n = 400,%)	(2n = 400,%)			
C A C A T A T G	4.00 (0.010)	7.00 (0.018)	—		—
C A C A T G T G	0.00 (0.000)	1.00 (0.003)	—		—
C A C G A A C G	2.00 (0.005)	6.09 (0.015)	—		—
C A C G A A T G	0.00 (0.000)	6.02 (0.015)	—		—
C A C G T A C G	4.06 (0.010)	2.03 (0.005)	—		—
C A C G T A T A	4.00 (0.010)	3.00 (0.008)	—		—
C A C G T A T G*	297.94 (0.745)	275.81 (0.690)	4.347	0.037	1.540 [1.024~2.314]
C A C G T G T G	0.00 (0.000)	3.05 (0.008)	—		—
C A T G T A T G	4.00 (0.010)	6.00 (0.015)	—		—
C G C A T A T G	4.00 (0.010)	2.00 (0.005)	—		—
C G C G T A T G	4.00 (0.010)	4.00 (0.010)	—		—
T A C A T A T G	2.00 (0.005)	2.00 (0.005)	—		—
T G T A A A C A	9.98 (0.025)	4.94 (0.012)	—		—
T G T A A A T A	2.02 (0.005)	2.02 (0.005)	—		—
T G T A A G C A*	46.96 (0.117)	66.92 (0.167)	4.347	0.037	0.650 [0.432~0.976]
T G T A A G T A	4.04 (0.010)	3.01 (0.008)	—		—
T G T A T A T A	0.00 (0.000)	4.09 (0.010)	—		—
T G T A T G C A	1.00 (0.003)	1.02 (0.003)	—		—
C A C G A G C G	6.00 (0.015)	0.00 (0.000)	—		—
C G C A A G T G	1.00 (0.003)	0.00 (0.000)	—		—
T A T A T A T G	2.00 (0.005)	0.00 (0.000)	—		—
T G C A A G T G	1.00 (0.003)	0.00 (0.000)	—		—

Adjusted *P* value (*P* < 0.004) indicates statistical significance.

**Table 4:** Genotype and allelic distribution of rs l7222919 and rs9579646 in IS and control subjects.

Genotype	IS subjects (n,%)	Control subjects (n,%)	*P* value	*OR*(95% *CI*)
rs l7222919				
TT	525 (64.8)	486 (58.9)		1.000
TG	258 (31.9)	296 (35.9)	0.043	0.807 (0.656~0.993)
GG	27 (3.3)	43 (5.2)	0.031	0.581 (0.354~0.955)
allele				
T	1308 (80.7)	1268 (76.8)		1.000
G	312 (19.3)	382 (23.2)	0.007*	0.792 (0.669~0.937)
rs 9579646				
AA	185 (22.8)	179 (21.7)		1.000
AG	391 (48.3)	422 (51.2)	0.386	0.896 (0.700~1.148)
GG	234 (28.9)	224 (27.1)	0.939	1.011 (0.768~1.331)
A	761 (47.0)	780 (47.3)		1.000
G	859 (53.0)	870 (52.7)	0.865	1.012 (0.882~1.161)

The *P* value and *OR* (95% *CI*) were adjusted for confounding factors such as age, gender, hypertension, diabetes, smoking, drinking, TC and TG. *express the adjusted *P* value for significance *P* < 0.025.

**Table 5:** Detailed association of rsl7222919 with IS risk in IS and control groups under different genetic models.

Model	Case (n, %)	Control (n, %)	χ^2^	*P* value	*OR* (95% *CI*)
Dominant					
TG + GG	285 (35.2)	339 (41.1)	6.040	0.014*	0.778 (0.637–0.951)
Recessive					
GG	27 (3.3)	43 (5.2)	3.520	0.061	0.627 (0.384–1.025)
Additive					
TT	525 (64.8)	486 (58.9)			
TG	258 (31.9)	296 (35.9)	4.110	0.043	0.807 (0.656 –0.993)
GG	27 (3.3)	43 (5.2)	4.674	0.031	0.581 (0.354–0.955)

*express the *P* value for significance *P* < 0.025.

**Table 6:** Haplotpype analysis of rsl7222919 and rs9579646 in IS and control groups.

Haplotype	Case (2n = 1620,%)	Control (2n = 1650,%)	*P* value	*OR* (95% *CI*)
G-G	41 (2.5)	19 (1.2)	—	—
T-G	818 (50.5)	851 (51.5)	0.536	0.958 (0.835–1.098)
G-A	271 (16.7)	363 (22)	0.0001*	0.712 (0.598–0.848)
T-A	490 (30.3)	417 (25.3)	0.001*	1.282 (1.100–1.495)

Note. Haplotypes with frequency <0.03 not considered in this analysis. *Significant differences between control and case groups (P < 0.025).
